# Novel metabolic and physiological functions of branched chain amino acids: a review

**DOI:** 10.1186/s40104-016-0139-z

**Published:** 2017-01-23

**Authors:** Shihai Zhang, Xiangfang Zeng, Man Ren, Xiangbing Mao, Shiyan Qiao

**Affiliations:** 1 State Key Laboratory of Animal Nutrition, College of Animal Science and Technology, China Agricultural University, No.2 Yuanmingyuan West Road, Haidian District, Beijing, 100193 People’s Republic of China; 2 College of Animal Science, South China Agricultural University, Wushan Avenue, Tianhe District, Guangzhou, 510642 People’s Republic of China; 3 College of Animal Science, Anhui Science & Technology University, No. 9 Donghua Road, Fengyang, 233100 Anhui Province People’s Republic of China; 4 Animal Nutrition Institute, Key Laboratory of Animal Disease-ResistanceNutrition,Ministry of Education, Sichuan AgriculturalUniversity, Ya’an, Sichuan China

**Keywords:** Amino acid transporters, Glucose transporters, Gut health, Immunity, Lipolysis, Mammary health, Meat quality, Milk production

## Abstract

It is widely known that branched chain amino acids (BCAA) are not only elementary components for building muscle tissue but also participate in increasing protein synthesis in animals and humans. BCAA (isoleucine, leucine and valine) regulate many key signaling pathways, the most classic of which is the activation of the mTOR signaling pathway. This signaling pathway connects many diverse physiological and metabolic roles. Recent years have witnessed many striking developments in determining the novel functions of BCAA including: (1) Insufficient or excessive levels of BCAA in the diet enhances lipolysis. (2) BCAA, especially isoleucine, play a major role in enhancing glucose consumption and utilization by up-regulating intestinal and muscular glucose transporters. (3) Supplementation of leucine in the diet enhances meat quality in finishing pigs. (4) BCAA are beneficial for mammary health, milk quality and embryo growth. (5) BCAA enhance intestinal development, intestinal amino acid transportation and mucin production. (6) BCAA participate in up-regulating innate and adaptive immune responses. In addition, abnormally elevated BCAA levels in the blood (decreased BCAA catabolism) are a good biomarker for the early detection of obesity, diabetes and other metabolic diseases. This review will provide some insights into these novel metabolic and physiological functions of BCAA.

## Background

The branched chain amino acids (BCAA: leucine, isoleucine, and valine) are essential amino acids and must be obtained from the diet. BCAA not only act as building blocks for tissue protein (accounting for 35% of the essential amino acids in muscle) [1], but also have other metabolic functions [1]. Among the three BCAA, leucine earns the greatest reputation for its specific function in activation of the mTOR signaling pathway. Since the 1970s, the role of leucine in enhancing protein synthesis has been reported both in vitro and in vivo [2, 3]. More recently, BCAA have been extensively used as performance-enhancing supplements for body builders and fitness enthusiasts [4, 5]. Besides playing a vital role in protein metabolism, a variety of physiological and metabolic functions have been reported for BCAA. For instance, BCAA were reported to increase the secretion of insulin [6]. However, increased level of plasma BCAA have also been reported to lead to insulin resistance or type 2 diabetes mellitus. One possible mechanism for this is that persistent activation of mTOR signaling pathway uncouples the insulin receptor from insulin receptor substrate 1 [7]. Another possible mechanism is accumulation of toxic BCAA metabolites (caused by abnormal BCAA metabolism) may trigger mitochondrial dysfunction which is associated with insulin resistance [8].

More recently, BCAA have been reported to participate in lipolysis, lipogenesis, glucose metabolism, glucose transportation, intestinal barrier function and absorption, milk quality, mammary health, embryo development, and immunity [9–14]. In addition, levels of BCAA in the body can act as a biomarker for the early detection of chronic diseases in humans [15]. The main objective of this review is to provide insights into new developments in BCAA research as well as their implications for both animal husbandry and human health.

### Metabolism of BCAA

**Fig. 1:**
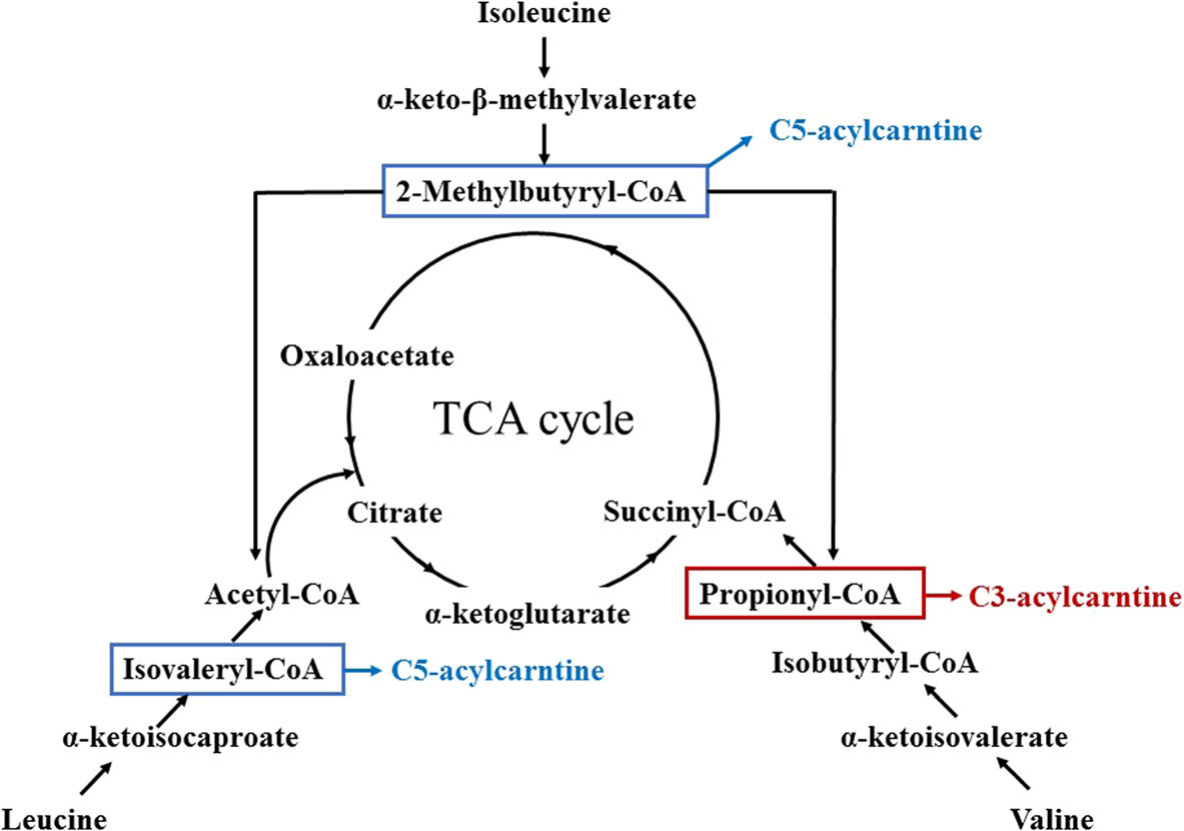
Pathway of branched chain amino acid catabolism. BCAA are catabolized to acetyl-CoA and/or succinate-CoA and subsequently enter the TCA cycle. The main steps of the catabolic reactions (transamination by BCAT and decarboxylation by BCKD) are shown. With the help of BCAT, BCAA are catabolized into branched-chain α-ketoacids which are subsequently decarboxylated by BCKD. Finally, all the BCAA metabolites are catabolized by a series of enzyme reactions to final products and enter the TCA cycle

The metabolism of BCAA is well established. However, this should be addressed before we start looking into the detail functions of BCAA as this can provide the reader with a better understanding of this paper. BCAA are not degraded directly in the liver and most of them are available for metabolism in skeletal muscle and other tissues. However, the liver can oxidize BCAA after they are converted into α-ketoacids in other tissues [16]. The main steps of BCAA catabolism are listed below (Fig. 1). Firstly, with the participation of branched-chain aminotransferase (BCAT), BCAA are converted into branched-chain α-ketoacids (leucine to α-ketoisocaproate, valine to α-ketoisovalerate, and isoleucine to α-keto-β-methylvalerate) by removing their amino group. Subsequently, branched-chain α-ketoacids are decarboxylated by branched-chain α-ketoacid dehydrogenase (BCKD). Finally, these BCAA metabolites are catabolized by a series of enzyme reactions to final-products (acetyl-CoA from leucine, succinyl-CoA from valine, and both acetyl-CoA and succinyl-CoA from isoleucine), which enter the TCA cycle.

### BCAA and fatty acid metabolism

In humans, consumption of diets with an increased protein and reduced carbohydrate content enhances weight loss with greater loss of body fat and less loss of lean body mass [17, 18]. In recent years, BCAA have been considered as novel therapeutic tools for controlling obesity and its related metabolic disorders, such as diabetes and insulin resistance, by enhancing exercise performance, regulating the composition of body protein and its properties, and controlling glucose tolerance, which are all related to improved health and fitness [16].

Studies in mice have indicated that diet-induced obesity mice in an isoleucine treatment (final concentration of 2.5% isoleucine in drinking water) had almost 6% lower body weight gain and 49% less epididymal white adipose tissue mass compared with the control treatment, with higher levels of hepatic protein CD36/fatty acid translocase, PPARα, and uncoupling protein (UCP) 2 and muscular levels of UCP3 [13]. Similarly, when dietary energy was restricted, leucine supplementation was found to increase fat loss and enhance muscle protein synthesis [19].

Interestingly, several studies have reported that supplying animals with a BCAA deficient diet increased lipolysis. Studies in mice discovered that feeding a leucine-deficient diet for 7 d suppressed lipogenesis in the liver [20] and also increased fat lipolysis in white adipose tissue [21]. Furthermore, isoleucine or valine deprivation also induced fat mass loss in mice [22]. Similarly, in female broiler chickens, low dietary BCAA levels reduced fatty acid synthesis and enhanced fatty acid-oxidation by up-regulating hepatic lipogenic gene ACCα and SCD-1 expression (ACCα is the enzyme for carboxylation of acetyl-CoA to malonyl-CoA which is the rate-limiting step for both synthesis and elongation of fatty acid synthesis while SCD-1 catalyzes the biosynthesis of monounsaturated fatty acids from dietary lipids) without affecting growth performance, and is likely mediated through the AMPK-mTOR-FoxO1 pathway [23].

A possible mechanism of a BCAA deficient diet in enhancing lipolysis is the activation of the GCN2 pathway. It was demonstrated that a leucine deficient diet resulted in reduction of food intake and weight lost in both GCN2^+/+^ and GCN2^−/−^ mice, but only resulted in loss of liver mass and abdominal adipose mass in GCN2^+/+^ mice [20].

Taken together, both insufficient or excessive levels of BCAA in the diet could be detrimental to lipid metabolism. Supplementation of BCAA increased acetyl CoA levels in cells which subsequently inhibited the activity of pyruvate dehydrogenase. The preference for the cellular energy source was shifted from carbohydrate to lipid. Also, BCAA up-regulates hepatic fatty acid translocation and fatty acid oxidation gene expression [13]. Compared with BCAA supplementation of the diet, feeding animals with a BCAA deficient diet dramatically reduces food intake by activating the GCN2 signaling pathway, which might participate in lipolysis (down-regulating lipogenesis genes or up-regulating lipolysis genes) in the liver and adipose tissue. At present, studies focusing on fatty acid metabolism are limited. Although some contradictions exist among different studies, the intimate relationship between BCAA metabolism and fatty acid metabolism cannot be denied and more research is needed in the future to explore these relationships.

### BCAA and glucose transportation

Accumulating evidence indicates a strong connection between amino acids and plasma glucose levels [24]. Branched chain amino acids have been demonstrated to strongly enhance glucose consumption and utilization [14]. In an animal oral glucose tolerance test, both isoleucine and leucine prevented a rise in plasma glucose concentrations, and the effect of isoleucine was greater than the other BCAA [14]. In a C2C12 myotubes experiment, both leucine and isoleucine stimulated glucose uptake [14]. Nishitani et al*.* [25] and Doi et al*.* [26] observed similar results showing that isoleucine participated in plasma glucose uptake in the rat. A hypothesis for the mechanism through which isoleucine and leucine regulate the serum glucose levels might be due to an increase in muscle glucose uptake, whole body glucose oxidation and a decrease in hepatic gluconeogenesis [27].

The fact that BCAA enhance glucose uptake with activation or up-regulation of glucose transporters has been widely demonstrated [14, 25]. Leucine increases glucose uptake by up-regulating the translocation of GLUT4 and GLUT1 in rat muscle [25]. Similarly, another experiment reported that leucine enhances the expression of GLUT4 glucose transporter and 2-deoxyglucose uptake in C2C12 cells [14]. Scientists suggest two hypotheses to interpret the mechanism through which leucine regulates muscular glucose transporters. Firstly, leucine enhances translocation of GLUT1 and GLUT4 by up-regulating insulin levels [28–30]. Secondly, leucine increases glucose uptake in skeletal muscle via the PI3K and PKC signaling pathways [14] both of which are associated with GLUT4 translocation [31].

**Fig. 2:**
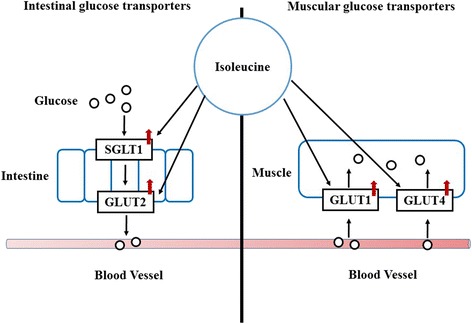
Isoleucine up-regulates intestinal and muscular transporters. GLUT1 and GLUT4 are vital glucose transporters in muscle. SGLT1 and GLUT2 are important glucose transporters in the small intestine. Isoleucine could potentially increase muscle growth and intestinal development and health by up-regulating the protein expression of GLUT1 and GLUT4 in muscle and enhancing the expression of SGLT1 and GLUT2 in the small intestine

Compared with leucine, research focusing on the mechanism through which isoleucine acts is limited. Recent studies done in our lab demonstrate that feeding weanling pigs an isoleucine deficient diet down-regulates the protein expression of GLUT1 in red muscle and GLUT4 in red muscle, white muscle and intermediate muscle (Fig. 2) [32]. Furthermore, our experiments showed that an isoleucine deficient diet suppresses the expression of intestinal glucose transporter SGLT-1 in the duodenum, jejunum and ileum and GLUT2 in the duodenum and jejunum (Fig. 2). The function of isoleucine in enhancing glucose uptake and muscular glucose transporter expression (GLUT1 and GLUT4) was also demonstrated in C2C12 myotubes in our study. However, the underlying mechanisms through which it functions are still unknown.

Collectively, BCAA regulate the expression and translocation of muscular or intestinal glucose transporters through insulin-dependent or insulin-independent ways. These findings have important implications in that BCAA could enhance muscle growth and intestinal development by increasing the local glucose uptake for animals and humans.

### BCAA and protein synthesis

**Fig. 3:**
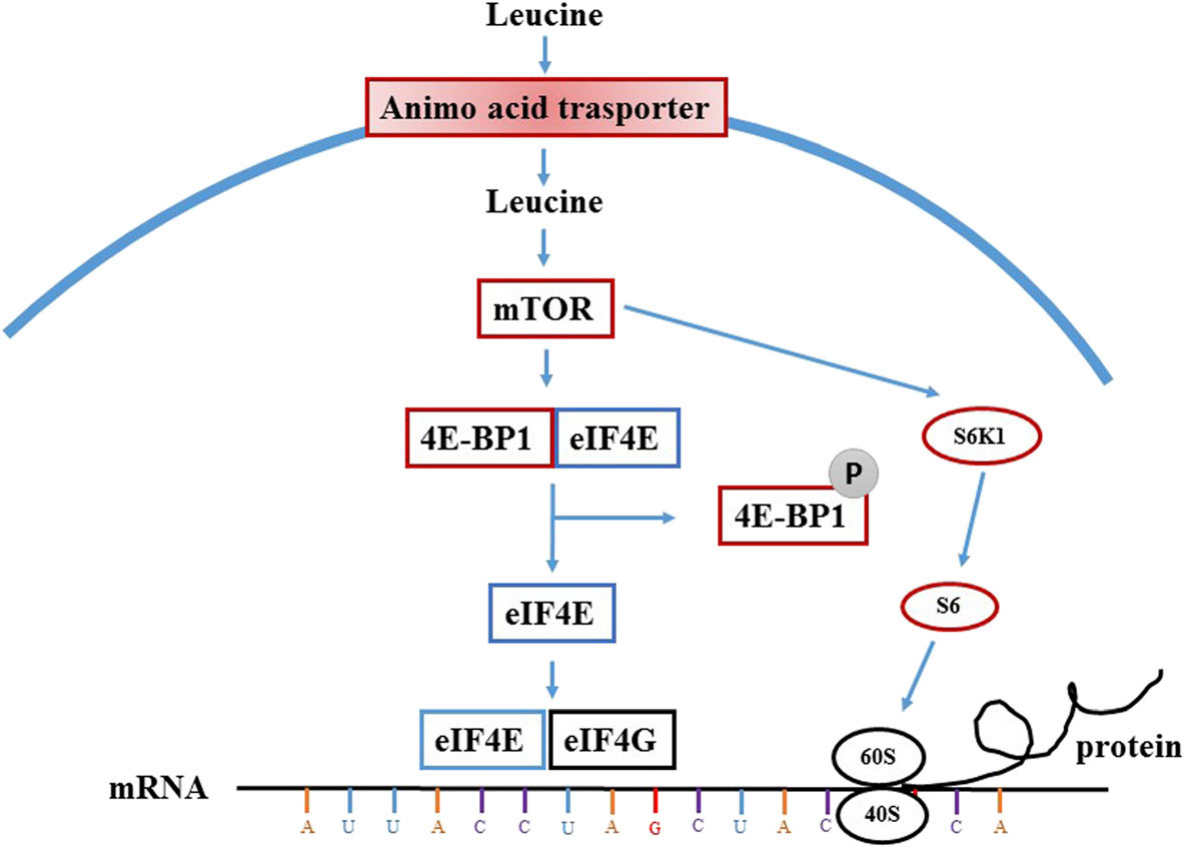
Leucine increases protein synthesis by activation of the mTOR signaling pathway. Leucine enhanced muscle synthesis via the mammalian target of rapamycin (mTOR) pathway leading to phosphorylation of its downstream target proteins, eukaryotic initiation factor 4E-binding protein (4E-BP1) and p70 ribosomal S6 kinase 1 (S6K1). Under unphosphorylated conditions, 4EBP1 tightly binds to eIF4E, forming the inactive eIF4E · 4EBP1 complex. During anabolic conditions, mTORC1 induces the phosphorylation of 4EBP1, resulting in the dissociation of eIF4E from the inactive complex and allowing eIF4E to form an active complex with eIF4G. The process of association of eIF4E with eIF4G is obligatory for the binding of the 43S pre-initiation complex with mRNA. S6K1 is another mTORC1 substrate that participates in the regulation of mRNA translation. This kinase plays an important role in the regulation of terminal oligopyrimidine mRNA which is responsible for the translation of proteins involved in the protein synthetic apparatus

Since 1999, Joshua C. Anthony, the pioneer in leucine functional research, conducted a series of experiments regarding the effects of leucine on muscle protein synthesis and its underlying mechanisms (Fig. 3). Firstly, his team observed that leucine stimulates the recovery of skeletal muscle protein synthesis after exercise, independent of increased plasma insulin [33]. Their studies also revealed that leucine enhances muscle protein synthesis via the mammalian target of rapamycin (mTOR) pathway leading to phosphorylation of its downstream target proteins, eukaryotic initiation factor 4E-binding protein (4E-BP1) and p70 ribosomal S6 kinase 1 (S6K1) [34, 35]. Since then, many experiments have been conducted which strongly support their results [5, 36]. Leucine has been shown to stimulate muscle protein synthesis in rats [19, 37, 38], pigs [39–41] and humans [5, 42, 43]. The team of Teresa A. Davis evaluated the function of leucine in neonates. They found leucine has unique anabolic properties and the supplementation of leucine or its metabolites α-ketoisocaproic acid and β-hydroxy-β-methylbutyrate strongly increase muscle protein synthesis in neonates [44–46]. Supplementation of leucine in a protein deficient diet had a strong positive connection to protein synthesis [47]. Interestingly, some studies reported that supplementation of leucine in a chronically restricted protein and energy diet only enhanced mTOR pathway activation without increasing muscle protein synthesis in neonatal pigs [48]. This indicates that the stimulation of protein synthesis by leucine is dependent on the availability of other amino acids [49].

Recently, the synergistic effect between leucine and leptin, and the effect of leucine on meat quality has been reported by our lab. In our study, we found that leucine stimulated the expression of leptin and its muscular receptor [50]. In addition, the combination of leptin and leucine synergistically regulated protein metabolism in skeletal muscle both in vitro and in vivo [51]. In an experiment with finishing pigs, we found that supplementation with leucine (1.25%) could enhance pork texture by enhancing protein deposition and improving meat quality [52]. Similarly, another experiment in finishing pigs reported that leucine addition increased juiciness accompanying the increase in intramuscular fat content occurring with a protein deficient diet [53]. However, supplementation of excess leucine (3.75%) in the diet changes plasma amino acid-derived metabolites, which may limit the use of high Leu diets to treat muscle atrophy [54]. Therefore, a high dose of leucine could be toxic and finding a suitable supplementation level of leucine is vital for future use.

In conclusion, the effect of leucine in increasing protein synthesis via the mTOR signaling pathway is widely known, the function of which might be enhanced by leptin. A reasonable supplementation level for leucine could improve meat quality.

### BCAA and feed intake

The function of central leucine infusion on feed intake inhibition has been demonstrated in many studies [55, 56]. The mTOR signaling pathway plays a vital role in the brain to detect nutrient availability and regulate energy balance [57]. In an experiment with rats, Cota et al*.* [57] demonstrated that mTOR signaling is controlled by energy status in specific regions of the hypothalamus and colocalizes with neuropeptide Y and proopiomelanocortin neurons in the arcuate nucleus. However, the function of leucine on feed intake is different when leucine is supplied in the diet. Many experiments demonstrated that extra supplementation of leucine in the diet not increase feed consumption in animals [58–61]. The divergent results caused by oral or central leucine supplementation might be explained by the capacity of leucine to cross the blood–brain barrier and reach the central neural system.

Although extra supplementation of BCAA in the diet does not further increase the feed intake, the function of a BCAA-deficient diet in down-regulating feed intake can not be ignored. Gloaguen et al*.* [62] conducted a trial to test if feed intake was affected after ingestion of Val- and Val + diets with an excess of Leu. They found that prior ingestion of the Val- test diet resulted in a 14% reduction in feed intake compared with Val + test meal. Zhang et al*.* [63] reported that feeding piglets with 17% crude protein BCAA-deficient diet significantly decreased feed intake by 42%. As BCAA are essential amino acids for animals, the reduction of feed intake could be interpreted as a function of the unbalance essential amino acid levels in the serum, which are regulated with the activation of GCN2 signaling pathway [64].

### BCAA and mammary function

A significantly increased whole-body BCAA catabolism has been observed during lactation compared with non-lactating counterparts [65, 66]. BCAA catabolism could enhance the syntheses of glutamate, glutamine, aspartate, alanine, and asparagine in the mammary gland and increase the production of milk for suckling neonates [11, 12]. In addition, the major leucine transporter LAT1 is a limiting factor for the synthesis of glutamate and aspartate in mammary tissue [67]. Concurrently, the activity of BCAT and BCKD (two vital enzymes in BCAA catabolism) are increased in mammary tissue during lactation [68], which might be caused by reductions in insulin and growth hormone or increases in cortisol and glucagon [69].

Accumulating evidence indicates the obvious connection among BCAA, milk production and neonatal piglet performance. An experiment in sows demonstrated that supplementation of BCAA in the diet (Control group: 16% CP vs. Treatment group: 23% CP + BCAA) increased milk protein secretion but not milk yield [70]. In early lactation in fistulated dairy cows, compared with abomasally infused EAA ones, omission of leucine or BCAA decreased protein yield about 12% and 21%, respectively [71]. A recent study found that increasing the total dietary Val:Lys ratio from 0.84:1 to 0.99:1 increased milk concentrations of isoleucine and valine [72]. These results indicate that the level of BCAA (especially leucine) in the diet plays a vital role in determining milk protein percentage. The underlying mechanism for a deficiency of BCAA impairing milk protein production is due to the deactivation of mTORC1-mediated up-regulation of eIF2Bε and eIF2α abundance [73]. However, another 2 × 2 × 2 factorial study in sows (two levels of valine (0.80 and 1.20%), isoleucine (0.68 and 1.08%), and leucine (1.57 and 1.97%)) found that only supplementation of valine tended to increase milk nitrogen, but not isoleucine or leucine [74]. For neonatal piglets, this research found that increasing dietary valine level from 0.8% to 1.2% in sows is vital for increasing litter weaning weight [74].

**Fig. 4:**
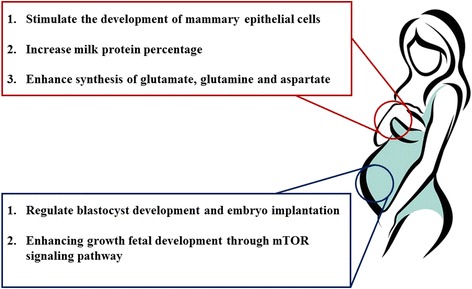
Branched chain amino acids regulate mammary function and embryo development. BCAA play a vital role in mammary function and embryo development mainly in the synthesis of other conditional amino acids and activation of the mTOR signaling pathway

The function of BCAA on milk production might be manipulated via the mammary cells (Fig. 4). In mammary epithelial cells, BCAA could stimulate their growth and proliferation, enhance their functional differentiation and increase their longevity [75]. Both leucine and isoleucine have been shown to enhance the fractional protein synthesis rates in bovine mammary cells with the phosphorylation of mTOR and rpS6, or mTOR, S6K1, and rpS6 respectively [76], while decreasing the abundance of proteasome protein, ubiquitinated protein, and the rate of protein degradation [77]. The mechanism through which valine functions is still unknown.

These novel findings not only advance our understanding of BCAA regulation of lactation but also provide a new strategy to improve milk production by livestock and humans.

### BCAA, blastocyst development and fetal growth

Early embryo growth and development of the fetus depend entirely on maternal nutrition. Alterations in fetal health are strongly associated with the development of chronic diseases later in adult life [78]. BCAA have been implicated as one of the vital elements in fetal development. Compared with women with healthy fetuses, pregnant women with fetuses with intrauterine growth retardation (IUGR) have lower plasma concentrations of BCAA in the umbilical artery and vein [79]. Protein synthesis is important for early embryo development. Among all the BCAA, leucine is the most important as it stimulates protein synthesis in skeletal muscle and other tissues through the mTOR signaling pathway [80]. Supplementation of BCAA (1.8% L-Leu, 1.2% L-Val, and 1% L-Ile) relieves IUGR syndrome induced by a low-protein maternal diet through activation of the mTOR signaling pathway [10]. Furthermore, a BCAA-supplemented diet is reported to improve the gene and protein expression of IGF-1 and IGF-2 in fetal liver which could ameliorate fetal growth restrictions [79]. However, another study found L-leucine (at doses of 300 or 1,000 mg/kg body weight) administered orally during organogenesis did not affect the outcome of pregnancy. in rats [81]. The conflict in results might be caused by different dietary leucine concentrations and BCAA combinations. It is apparent that leucine up-regulates fetal growth by the enhancement of protein synthesis and secretion of hormones.

Besides being necessary for the development of the embryo, amino acids enhance embryo implantation by improving blastocyst quality. Researchers have found that amino acids are necessary for cultivating mouse embryos in vitro [82] which might be because of the embryonic requirement for AA as basic nutrients. Subsequently, amino acids are proved to induce trophectoderm motility and mouse embryo implantation via activation of the mTOR signaling pathway [83–85]. As one of the most vital roles for leucine is activating the mTOR signaling pathway, this indicates that leucine might participate in blastocyst development. Recently, it has been reported that up-regulation of leucine transporter SLC6A14 induces blastocyst activation [86, 87]. All this evidence indicates that leucine has an important function in blastocyst development. However, the optimal levels of BCAA required during pregnancy and lactation for animals and humans are still unknown.

### BCAA and gut function

In addition to glutamine and asparagine, large amounts of BCAA are consumed and oxidized in the intestine. BCAA were catabolized in jejunal mucosal cells with a high activity of cytosolic BCAT and about 30% of the BCAA-derived branched-chain α-ketoacid were decarboxylated by BCKD [88, 89]. Several studies have found that BCAA participates in intestinal amino acid transporter expression [90]. Supplementing 1.4 g L-leucine/kg body weight to breast-fed neonates improves intestinal development and increases the expression of neutral amino acid transporters (ATB^0,+^, B^0^AT1 and b^0,+^AT) [90]. In a study conducted in our lab, we demonstrated that meeting BCAA requirement (supplementing 0.1% L-Leu, 0.34% L-Val, and 0.19% L-Ile in 17% crude protein diet) is necessary for maintaining intestinal health and amino acid transporter expression [63], the latter of which might be through the PI3K/Akt/mTOR and ERK signaling pathways [91]. Interestingly, we found that leucine not only increased the expression of neutral amino acid transporters (ASCT2, rBAT and 4F2hc), but also cationic amino acid transporters (CAT1) which emphasizes the importance of BCAA in intestinal nutrient absorption.

Besides regulating intestinal amino acid transporter expression, BCAA also have an intimate connection with other intestinal functions. Elevating dietary leucine level from 1.37% to 2.17% enhanced the intestinal development of broilers through the mTOR signaling pathway [92]. Concurrently, Mao et al*.* [93] demonstrated that extra dietary 1% leucine supplementation alleviated a decrease in mucin production and goblet cell numbers in the jejunal mucosa of weaned pigs, which possibly occurs via activation of mTOR signaling. Similarly, the role of leucine (increasing leucine level from 0.71% to 1.33%) in maintaining gut health (enhancing tight junction) was also demonstrated in fish [94].

BCAA can be utilized by bacteria in the lumen of the gut [95]. Based on the 24-h disappearance rates of amino acids in different intestinal segments, Dai et al*.* [95] divided AA into three groups (high, medium or low disappearance rate groups), and found that leucine belongs to the high disappearance rate group while isoleucine and valine belong to the medium disappearance rate group. This is important evidence that BCAA participate in bacterial metabolism indicating they might participate in the regulation of intestinal microbial species and diversity. More research needs to be done to elucidate the detailed changes in these microbial species.

Collectively, most of the studies still focus on the functions of leucine but not valine or isoleucine in the intestine. However, the high expression of BCAT and BCKT in the intestine indicates the strong connection between BCAA and intestinal function.

### BCAA and immune function

People noticed the effects of BCAA on the immune system 10 yr ago because immune cells oxidize BCAA as fuel sources and incorporate BCAA as the precursors for the synthesis of new immune cells, effector molecules, and protective molecules [9]. Lack of BCAA in the diet impairs many aspects of immune function and increases susceptibility to pathogens. A recent study showed that a daily 12 g BCAA oral supplementation improved phagocytic function of neutrophils and NK activity of lymphocytes in cirrhotic patients [96]. After a BCAA enriched solution was infused into patients with rectal cancer, their immune status was improved with increased CD4+, CD4+/CD8+ and IL-2R [97]. Similarly, 12 g/d of BCAA (6 g/d L-Leu, 2 g/d L-Iso and 4 g/d L-Val) supplementation blunted the neutrophil response to intense cycling training, which might benefit immune function during a prolonged cycling season [98]. In animal husbandry, supplementing BCAA (0.1% L-Leu, 0.34% L-Val, and 0.19% L-Ile) in a 17% crude protein diet was shown to improve intestinal immune defense functions with an increase of intestinal immunoglobulins (IgA and sIgA) in weaned piglets [99]. In contrast, some studies also reported that BCAA mixture supplementation (600 mg/kg body weight/day, consist of 46% leucine, 28% valine, and 23% isoleucine) could not ameliorate the impaired function of macrophages induced by strenuous exercise in rats [100].

In recent years, there has been growing interest in the role of isoleucine, leucine and valine in immune function (Table 1). Notably, concentrations of isoleucine have a strong correlation with the excretion of *β*-defensin. 25 or 50 μg/mL isoleucine increases the mRNA and protein expressions of *β*-defensin 1, 2 and 3 in IPEC-J2 cells [101]. Also, treating patients with 250 μg of intratracheal L-isoleucine every 48 h is considered as a novel immunotherapy in tuberculosis as it induced a significant increase of *β*-defensins 3 and 4 associated with decreased bacillary loads and tissue damage [102]. This inducing function of isoleucine might be associated with the G-Protein Coupled Receptor and ERK signaling pathways [103]. Additionally, proximally 2% dietary isoleucine could enhance intestinal immunity in juvenile Jian carp and innate immunity in olive flounder [104, 105]. Similar functions in regulation of the immune response and antioxidant status in the head kidney were also observed in fish fed about 1.3% isoleucine [106]. In contrast to isoleucine, leucine regulates the immune system mainly through the mTOR signaling pathway. mTOR plays a vital role in the regulation of the innate and adaptive immune responses and also several immune functions like promoting differentiation, activation, and function in T cells, B cells and antigen-presenting cells [107, 108]. For instance, a reasonable dose of leucine (40 mg/mL) provides enhanced protective immunity against mucosal infection with herpes simplex virus type 1 [109]. Leucine deficiency could impair the immune status, up-regulate pro-inflammatory cytokines and down-regulate anti-inflammatory cytokines of grass carp (Ctenopharyngodon idella) by the NF-κB and TOR signaling pathways which was reversed by optimum leucine supplementation [94]. Recently, an in vitro experiment found that an increased extracellular concentration of BCAA, especially valine (800 nmol/mL), could improve the dendritic cell function in cirrhotic patients [110]. In addition, valine deficiency (less than 1.45%) decreased growth and intestinal immune status in young grass carp (Ctenopharyngodon idella) by increasing pro-inflammatory cytokines (IL-8 and TNF-α) and decreasing anti-inflammatory cytokines (IL-10 and TGF-β1) which might be caused by changes in the NF-κB and mTOR signaling pathways [111]. However, some studies found there was little effect of valine (increase from 0.64% to 0.87%) on innate or adaptive immunity for broilers [112]. Although still unclear, all these studies are evidence that BCAA may function in improving health and preventing infectious diseases in animals and humans by regulating the immune system.

**Table 1:** Branched chain amino acids and immune function

Amino acid	Regulation of immune function
BCAA Mix	● ↑ fuel sources for immune cells ● ↑ immune function of neutrophils and lymphocytes ● ↑ CD4+, CD4+/CD8+ ● ↑ intestinal immunoglobulins
Isoleucine	● ↑ excretion of *β*-defensin
Leucine	● ↑ regulation of innate and adaptive immune responses ● ↑ pro-inflammatory cytokines ↓ anti-inflammatory cytokines
Valine	● ↑ dendritic cell function ● ↑ pro-inflammatory cytokines ↓ anti-inflammatory cytokines

### BCAA, a biomarker for early pathogenesis of chronic diseases

Obesity is strongly associated with the risk of developing a number of chronic diseases including diabetes, gallstones, hypertension, heart disease and stroke [113]. Scientists have attempted to find biomarkers which can connect the incidence of these diseases via metabolomics. Alterations in their metabolism may play a vital role in the early pathogenesis for humans. Recently, the relationship between metabolomics and obesity (insulin-resistant) was revealed by a series of studies [114]. Newgard et al*.* [15] found that some major components obtained from obese (insulin-resistant) versus lean (insulin sensitive) subjects were different, including long-chain fatty acids, ketone metabolites and medium-chain acylcarnitines, but surprisingly, the component which was most explicitly associated with insulin sensitivity was not the lipid related components mentioned above, but rather was comprised of the BCAA, the aromatic amino acids, C3 and C5 acylcarnitines (Fig. 1), as well as glutamate and alanine. Concurrently, recent metabolomics conducted with 2,422 normoglycemic individuals for 12 yr showed a strong association between metabolite profiles (branched-chain and aromatic amino acids) and future diabetes [115]. In addition, subsequent reports showed that leucine and isoleucine levels (but not valine) are correlated with insulin resistance and blood glucose levels which indicates that analyzing BCAA separately might be better for understanding the association between BCAA and obesity [116]. In study involving twins, BCAA catabolism of the obese was down-regulated compared with lean co-twins in the adipose tissue [117], which could be caused by a decrease of BCATm and BCKD E1α protein concentrations (first two enzymatic steps of BCAA catabolism)[118]. Studies in animals also strongly support the findings in humans. BCAA metabolism is weak in diabetic mice compared with non-diabetic mice [119]. In addition, high running capacity rats express more BCAA degradation and fatty acid metabolism genes than low running capacity rats [120]. Taken together, elevated BCAA levels in blood (decreased BCAA catabolism) are associated with obesity, diabetes, and other risk factors for metabolic diseases and are a good biomarker for early pathogenesis of these diseases in humans.

## Conclusion and perspectives

BCAA are essential amino acids for animals and humans not only because they cannot be synthesized in the body but also because they display remarkable metabolic and regulatory roles. In humans and animals, BCAA (especially leucine) enhance protein synthesis through the mTOR signaling pathway and now are considered as feed additives to regulate meat quality and are used as performance-enhancing supplements for body builders and fitness enthusiasts. Recently, novel metabolic and physiological functions of BCAA have been reported by scientists. BCAA are metabolic regulators not only in protein synthesis but also in lipid and glucose metabolism. They enhance mammary health, increase milk quality and help in early embryo implantation and development. They improve gut health and its local amino acid transporting ability. They enhance immunity by increasing the expression of *β*-defensin, up-regulating pro-inflammatory cytokines and down-regulating anti-inflammatory cytokines. Finally, they are biomarkers for early detection of chronic diseases like diabetes and insulin resistance in humans.

A growing body of evidence suggests that food has specific direct and indirect actions to activate intestinal receptors like a cocktail of ‘hormones’ [121]. This activation can increase the secretion of GI tract hormones like peptide YY (PYY), glucagon-like peptide 1 (GLP-1) and cholecystokinin (CCK) [122]. There are variety of receptors in the GI tract for amino acids which have been discovered such as T1R1/T1R3, CaSR, GPCR6A and mGluR [123]. The activation of these receptors might participate in the regulation of food intake, proliferation of GI cells, small intestinal motility and neural reflexes [124]. However, the specific receptors for BCAA are still a mystery and wait to be discovered. Knowing the receptors of BCAA is vital for a better understanding of the physiological roles of BCAA.

In the future, with the help of high throughput functional genomics, metabolomics, and proteomics, the underlying functions of BCAA in gene, protein expression and metabolic regulation will be revealed. The effects of the BCAA on microbe numbers can be analyzed by 16S targeted sequencing and metagenomic sequencing. All of these techniques will help in interpreting the complex and inconsistent results obtained to date and largely expand our vision of the novel functions of BCAA in humans and animals.
